# Transcriptomic insights into the resistance mechanism of *Penaeus vannamei* against highly lethal *Vibrio parahaemolyticus*

**DOI:** 10.1038/s41598-025-96168-3

**Published:** 2025-04-18

**Authors:** Zhihao Huang, Yifei Liao, Jianrong Du, Zhongming Yang, Fang Li, Lingwei Ruan, Hong Shi

**Affiliations:** 1 State Key Laboratory Breeding Base of Marine Genetic Resources, Key Laboratory of Marine Genetic Resources of Ministry of Natural Resources, Third Institute of Oceanography, Ministry of Natural Resources, Fujian Key Laboratory of Marine Genetic Resources, No. 178 Daxue Road, Xiamen, 361005 Fujian People’s Republic of China; 2 Xiamen Xinrongteng Aquaculture Co., Ltd, Xiamen, 361005 People’s Republic of China; 3 School of Advanced Manufacturing, Fuzhou University, Quanzhou, 362251 People’s Republic of China; 4 Co-Innovation Center of Jiangsu Marine Bio-Industry Technology, Jiangsu Ocean University, Lianyungang, 222005 People’s Republic of China

**Keywords:** Highly lethal *Vibrio* disease, Transcriptomic analysis, *Penaeus vannamei*, *Vibrio parahaemolyticus*, Immunology, Microbiology, Molecular biology, Zoology, Pathogenesis

## Abstract

Highly lethal *Vibrio* disease (HLVD) caused by a virulent strain of *Vibrio parahaemolyticus* (*Vp*_HLVD_), which poses a significant threat to *Penaeus vannamei* post-larvae, leads to substantial mortality and economic losses. To address this challenge, researchers have recently isolated a highly disease-resistant strain of *P. vannamei* shrimp. However, the underlying mechanisms that could improve disease resistance require further investigation. Our study found that disease-resistant shrimp exhibited a remarkable ability to prevent *Vp*_HLVD_ invasion effectively. To unravel the genetic basis of this resistance, we conducted a transcriptomic analysis with susceptible and disease-resistant shrimp at various time points (0, 6, and 12 h) post-infection with *Vp*_HLVD_. Differential gene expression (DEGs) analysis of uninfected shrimp revealed that disease-resistant individuals displayed higher expression of immune-related genes and pathways compared to their susceptible counterparts. Simultaneously, they exhibited lower expression of *Vibrio* toxin-binding genes and *Vibrio* colonization gene, indicating enhanced defense mechanisms in the resistant shrimp. Upon *Vp*_HLVD_ infection, DEGs analysis also showed that susceptible shrimp attempt to mount a similar immune response as the disease-resistant shrimp during the early stages of infection. However, as the infection progresses, the defense strategies diverge between the two groups, with the peak of gene response occurring later in the disease-resistant shrimp. Our findings indicated that disease-resistant shrimp did not experience significant stress during the early stages of infection and are capable of effectively enhancing their immune response in the middle and late stages of the infection. In summary, our study enhanced the understanding of the mechanisms employed by disease-resistant shrimp to combat *Vibrio*, and would help to develop effective strategies for disease prevention and control, ultimately reducing the impact of HLVD on shrimp aquaculture.

**Supplementary Information:**

The online version contains supplementary material available at 10.1038/s41598-025-96168-3.

## Introduction

Vibriosis, a bacterial disease caused by *Vibrio* species, is a significant challenge to the shrimp aquaculture industry, impacting both production and trade worldwide^1^. These bacteria, which are naturally present in wild and farmed shrimp habitats^2,3^, can lead to severe diseases and even 100% mortality rates, and resulting in substantial economic losses^4,5^. While *Vibrio* infections initially tend to affect hatcheries, they can also cause devastating outbreaks in grow-out ponds^5^.

*Vibrio parahaemolyticus* is recognized as a key pathogen in shrimp farming. It is responsible for the outbreaks of early mortality syndrome (EMS) or acute hepatopancreatic necrosis disease (AHPND)^6,7^. In 2019, the emergence of the Highly lethal *Vibrio* disease (HLVD), also known as “glass post-larvae disease,” brought devastating consequences to the post-larval population of *Penaeus vannamei*. The root cause of this lethal affliction had been pinpointed to virulent strains of *V. parahaemolyticus*, specifically *Vp*_HLVD_^8,9^. Infection with *Vp*_HLVD_ has a severe detrimental effect on the hepatopancreas and midgut epithelial cells of shrimp, leading to significant tissue damage. Despite the apparent similarities in symptoms with AHPND due to *Vibrio* involvement, *Vp*_HLVD_ stands out for its significantly higher virulence highlighted by Yang et al.^8^. In *Vp*_HLVD_-affected farms, a high mortality rate of post-larvae, exceeding 90%, is witnessed within a period of 24–48 h, following the first manifestation of abnormality in the shrimp population^10^.

Recent studies have found that Tc toxin is the primary causative agent behind this high mortality of *Vp*_HLVD_^10^. Tc toxin was first identified in *Photorhabdus luminescens* and is a class of toxin that acts intracellularly^11^. It consists of three subunits: TcA, TcB, and TcC, forming a protein complex. The study on Tc toxin in *Yersinia* and *Photorhabdus* identified TcA is responsible for membrane penetration and toxin translocation through binding to target cells. Additionally, TcB forms a complex with TcC that contains the autoproteolytically cleaved toxic component. The binding of Tc toxin to target cells triggers the opening of internal channels and injection of toxin into host cells^12–15^.

In the quest to combat vibriosis in shrimp, extensive research has been explored to understand their immune mechanisms. Fibrinogen-related protein (FREP), a germline-encoded pattern recognition receptor, played a pivotal role in recognizing *Vibrio* infections^16^. Humoral immunity, with its protective mechanisms such as phenoloxidase system^17^, antimicrobial peptides like the anti-lipopolysaccharide factor that inhibited *V. parahaemolyticus* growth^18^, and haemolymphatic coagulation system^19^, exemplified by mannose-binding lectin^20^, contributes significantly to resistance. Additionally, cellular immunity demonstrated its strength through processes like apoptosis induction, such as the B cell lymphoma-2-related ovarian killer protein’s role in *Vibrio harveyi* infections^21^, and phagocytosis, where gene silencing of Rab proteins in shrimp hemocytes reduced *V. parahaemolyticus* uptake^22^. RNA interference also plays a crucial part in the immune response. Notably, signaling pathways, like the Toll pathway’s Spätzle and MyD88 proteins activated and the enhanced IMD pathway homologues during *Vibrio* infections, were vital for coordinating the immune response^23,24^. These findings provide valuable insights into shrimp’s defense mechanisms and underscore the need for further investigation to develop effective strategies for preventing and controlling *Vibrio* disease.

Recently, a highly disease-resistant strain of *P. vannamei* was isolated, displaying an impressive survival rate above 90% against *Vp*_HLVD_ infection. Building on this resistant post-larval population, a comprehensive transcriptomic analysis was conducted, comparing susceptible and resistant shrimp at different time points post *Vp*_HLVD_ infection. Furthermore, we analyzed the gene expression differences, with the aim of identifying key genes involved in the shrimp’s response to *Vp*_HLVD_ and understanding their functional roles. This study will contribute to a deeper comprehension of the interactions between shrimp and *Vibrio*, as well as the underlying resistance mechanisms, ultimately paving the way for the development of more effective strategies for controlling *Vibrio* infections in shrimp aquaculture.

## Materials and methods

### Experimental animals

Two unselected *P.* *vannamei* post-larvae strains (C3 and S) (referred to as susceptible shrimp) and two selected post-larvae strains resistant to *Vp*_HLVD_ (B13 and B20) (referred to as disease-resistant shrimp) were obtained from Xiamen Xinrongteng Aquaculture Co. Ltd. All post-larvae used in the experiments were acclimated at a density of 50 animals/L for 1–2 days in recirculating seawater at 21 °C with a salinity of approximately 11%.

All animal experiments in this study followed by the Animal Welfare and Use Committee of Third Institute of Oceanography, Ministry of Natural Resources.

### Mortality experiments and sample collection

For the purpose of artificial infection, 100 each of the susceptible shrimp from C3 and S strains and 100 each of the disease-resistant shrimp from B13 and B20 strains were used in the study. A stock solution of highly lethal *V. parahaemolyticus* was added into the culture water to make the final concentration of *Vp*_HLVD_ to be 2.5 × 10^5^ CFU/mL. At the same time, the survival rate of post-larvae was assessed through manual counting at 1-h intervals during the initial 12 h of infection and at 6-h intervals thereafter.

Two types of post-larvae were categorized into three groups (uninfected stage (0 h), the early infection (6 h), the middle and late infection (12 h)), and post-larvae samples were randomly collected at 0 h as well as at 6 and 12 h post *Vp*_HLVD_ infection. 9 individuals were collected at random from each strain in each group. The post-larvae samples were homogenized in TRIzol and stored at − 80 °C.

### LC–MS/MS analysis

Susceptible shrimp feces were lysed by mixing with 2 × SDS protein loading buffer (Solarbio, Beijing, China), and boiled for 10 min. The proteins in the samples were then separated using 10% SDS-PAGE gel electrophoresis, and the major protein bands were excised from the gel after staining with Coomassie Brilliant Blue. LC–MS/MS analysis was conducted on the major protein of post-larvae feces by Sangon Bioengineering (Shanghai) Co., Ltd. The raw data obtained was searched against the *P. vannamei* proteome reference database by ProteinPilot (V4.5).

### Total RNA extraction

Post-larvae total RNA was extracted using the TRIzol method (Invitrogen, USA) according to the manufacturer’s protocol, and the integrity of total RNA was assessed by 1% TAE gel electrophoresis. The concentration and purity of total RNA were detected using NanoDrop 2000 (Thermo Scientific, USA).

### Preparation for Illumina sequencing

The mRNA (with PolyA tail) was enriched by Oligo(dT) magnetic beads, and the mRNA obtained was randomly interrupted by divalent cations in Fragmentation Buffer. cDNA was synthesized using the fragmented mRNA as a template, and PCR amplification was carried out after performing end-repairing, connecting to sequencing junctions, and adding A tails to the cDNA. The resulting cDNA library underwent quality control before being sequenced using Illumina NovaSeq 6000 (Novozymes, Beijing, China).

### Illumina sequencing, transcriptome assembly, and functional annotation

The raw data were processed to obtain clean reads by filtering out duplicates, reads with low quality (where bases with Qphred ≤ 20 made up more than 50% of the read length), reads with adapters, and reads containing undetermined base information (N). Clean reads were spliced using Trinity (version 2.6.6) software to obtain reference sequences for subsequent analysis^25^. The left files (read1 files) from all libraries/samples were pooled into one big left.fq file, and the right files (read2 files) into one big right.fq file. Transcriptome assembly was then performed using Trinity, with min_kmer_cov set to 2 (default) and other parameters left at their default settings.

### Differentially expressed genes (DEGs) analysis

Expression levels of genes and transcripts were calculated using RSEM (RNA Seq by Ex pectation Maximization) software^26^. Differential expression analysis was conducted using DESeq2 (version 1.20.0), a tool that identifies differential expression in numerical gene expression data through a negative binomial distribution model. The *p*-value (padj) was calculated to control false discovery rate using the Benjamini and Hochberg method. A gene was deemed to be differentially expressed if its *p*-value was less than 0.05 and the absolute value of the log2 fold change was greater than 1. In such cases, the gene was classified as differentially expressed^27^.

Utilizing KOBAS (version 2.0.12) (http://bioinfo.org/kobas)^28^ and GOseq (version 1.10.0) (https://www.bioconductor.org/packages/release/bioc/html/goseq.html)^29^, we conducted KEGG pathway enrichment analysis and GO functional enrichment analysis on DEGs. This analysis was based on the hypergeometric distribution principle, and the DEGs identified from the significant difference analysis were annotated to the KEGG or GO database.

### cDNA synthesis

To detect Tc toxin, we used cDNA synthesis by TransStart® One-Step gDNA Removal and cDNA Synthesis SuperMix (TransGen Biotech, Beijing, China). Under RNase-free conditions, the system included 2 µg total RNA, 1 µl Random Primer (N9), 10 µl 2 × TS Reaction Mix, 1 µl TransStart® RT/RI Enzyme Mix, 1 µl gDNA Remover and RNase-free Water to 20 µl was incubated 42 °C for 15 min, 85 °C for 5 s.

To validate the transcriptome, genomic DNA was removed from the total RNA using DNase I (Takara). Subsequently, 2 µg of treated total RNA was incubated with oligo (dT)_18_ primer (Takara) at 70 °C for 10 min, followed by cDNA synthesis using M-MLV reverse transcriptase (Takara).

### Quantitative real-time PCR (qRT-PCR) analysis

For the detection of Tc toxin, primer design for the Tc toxin gene (TcA and TcB) was performed using Primer5 software (Table S1), and 16S rRNA was selected for the internal reference gene. qRT-PCR was conducted in triplicates for each sample using a total reaction system of 20 μl. This system included 10 μl of 2 × TransStart® Top Green qPCR superMix (TransGen Biotech, Beijing, China), 1 μl each of the Forward and Reverse primers, 6 μl of Nuclease-free Water, and 2 μl of template cDNA. The reaction program consisted of pre-denaturation at 94 °C for 30 s, followed by denaturation at 94 °C for 5 s, annealing at 56 °C for 15 s, and extension at 72 °C for 10 s. The mRNA levels were analyzed using the 2^−ΔΔCt^ method and statistically analyzed using Prism 5 software^30^.

The transcriptome results were validated using qRT-PCR, and 11 genes were selected from the DEGs obtained from the analysis and screening. Primers were designed for these DEGs using Primer5 software (Table S1), and the internal reference genes were Lv40S. qRT-PCR detection and analysis were performed as above.

## Results

### Evaluation of disease resistance in *P. vannamei* post-larvae

**Fig. 1:**
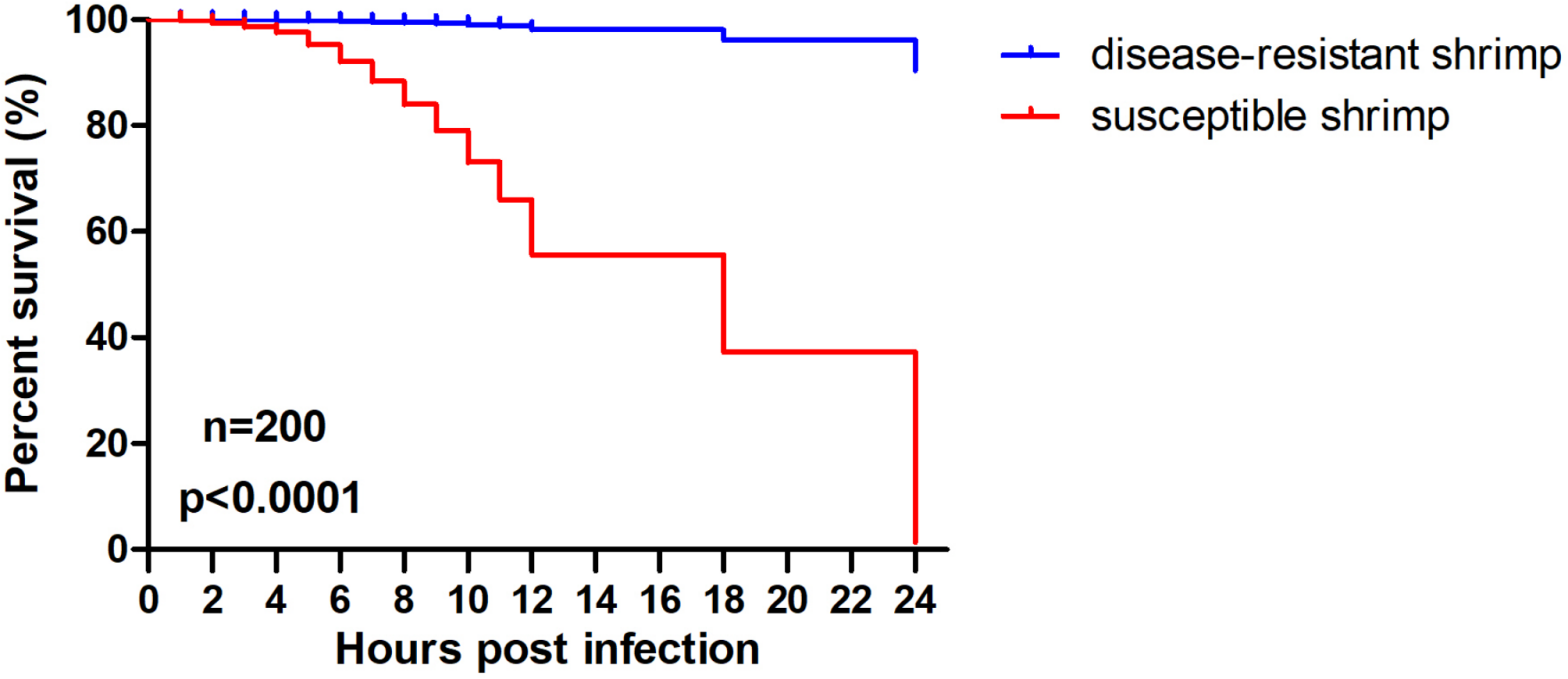
Survival rate statistics of disease-resistant and susceptible shrimp post-larvae. One hundred post-larvae from each group (C3, S, B13, B20) were immersed in seawater containing 2.5 × 10^5^ CFU/mL *Vp*_HLVD_, and their survival rates were determined at various time points. The data were analyzed statistically using a paired Student’s t-test, with a significant result (*p* < 0.0001).

The experimental setup began by acclimating both disease-resistant and susceptible shrimp post-larvae in a recirculating water system maintained at 21 °C and a salinity of approximately 11% for 1–2 days, allowing them to adapt to the environment condition. Following this adaptation period, *Vp*_HLVD_ was artificially introduced to the water column by adding a stock solution at a concentration of 2.5 × 10^5^ CFU/mL. Throughout the study, we closely observed and documented the survival dynamics of the shrimp. The results demonstrated a significant difference between disease-resistant (B13, B20) and susceptible shrimp (C3, S) in the response to *Vp*_HLVD_ infection. The disease-resistant shrimp exhibited a significant survival advantage, maintaining a low mortality rate and displaying robust vitality. In contrast, the susceptible shrimp exhibited clear pathologic signs, such as an empty digestive tract and pale or colorless hepatopancreas, and experienced a rapid decline in survival, with a mortality rate soaring to 96% within the 24 h (Fig. 1). This study contrasting vulnerability of shrimp populations to *Vp*_HLVD_, with the disease-resistant shrimp exhibiting superior resistance against lethal infections. In essence, the resistant shrimp exhibited evident advantages in combating *Vp*_HLVD_ infection.

### LC–MS/MS analysis of feces from susceptible shrimp

**Fig. 2:**
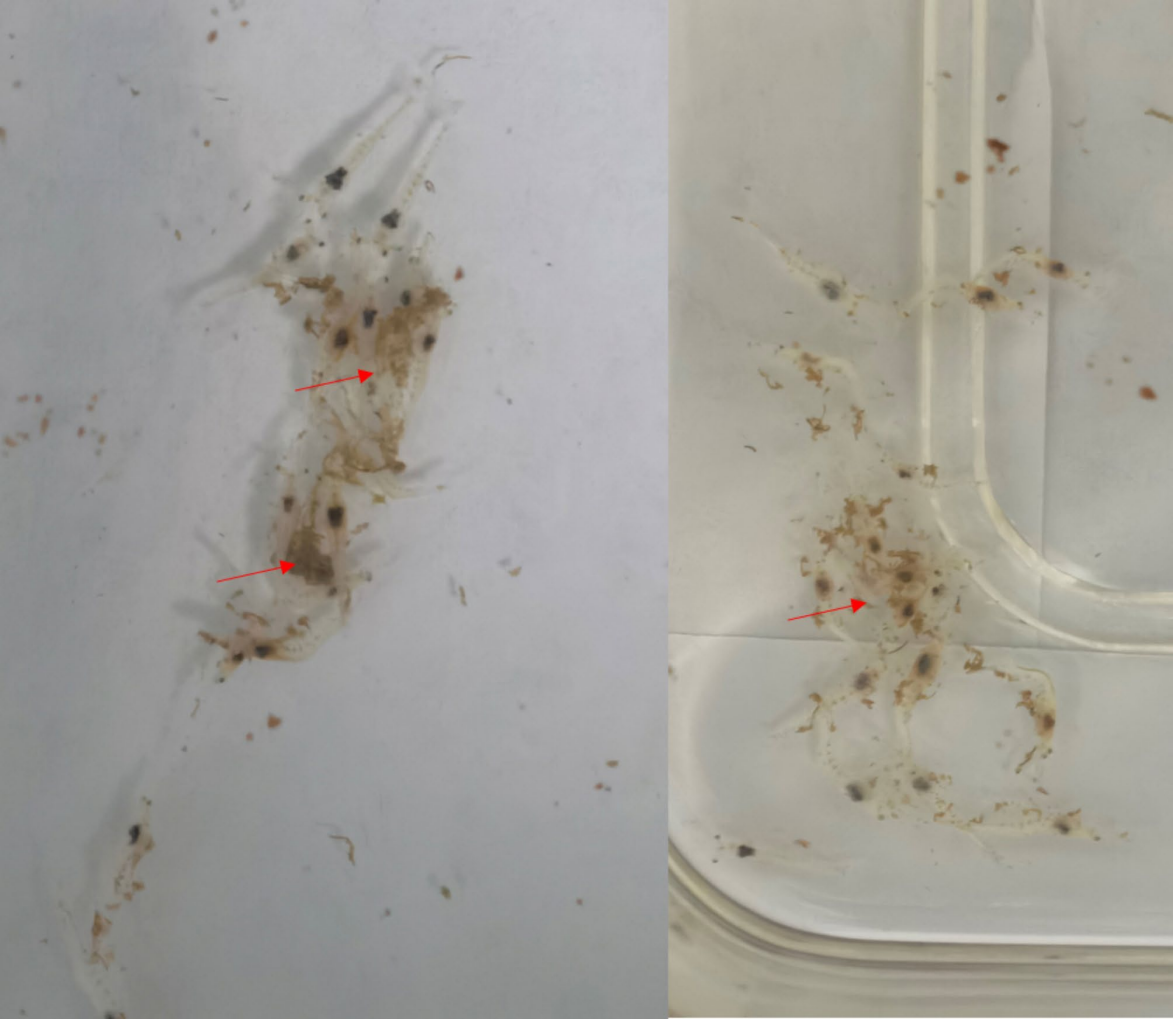
Fecal adhesion in susceptible shrimp. The red arrow indicates the adhesion of fecal matter to the shrimp’s body, a phenomenon specifically observed in susceptible shrimp.

We observed a fecal adhesion phenomenon in the susceptible shrimp (Fig. 2), which hindered their mobility and had an impact on their survival rate. To further investigate the fecal adhesion phenomenon, we collected feces and analyzed their compounds using LC–MS/MS, cross-referencing the raw data against the *P. vannamei* proteome reference database. We found that the identified proteins (Table 1), including Myosin heavy chain type 2, Putative nesprin-1, Fast-type skeletal muscle actin 15, Actin T2, and Tropomyosin Pen a 1.0102, were primarily cytoskeletal components. Conversely, we did not identify significant protein bands in the feces of the disease-resistant shrimp. This indicated that *Vp*_HLVD_ infection had a profound, serious impact on the digestive system of the susceptible shrimp, disrupting their structural integrity.

**Table 1:** LC–MS/MS analysis results of feces from susceptible shrimp.

GenBank accession number	Species	Description	Score	Coverage (%)	Number of matched peptides
ROT68165.1	*Penaeus vannamei*	Myosin heavy chain type 2	652	70	208
ROT63387.1	*Penaeus vannamei*	Hemocyanin subunit L3	888	54	253
ROT63387.1	*Penaeus vannamei*	Hemocyanin subunit L3	1133	61	288
ROT70123.1	*Penaeus vannamei*	Putative nesprin-1	1360	76	340
AUB30160.1	*Penaeus vannamei*	Fast-type skeletal muscle actin 15	943	71	234
ROT66127.1	*Penaeus vannamei*	Actin T2	1445	72	246
CAG5073674.1	*Metapenaeus ensis*	Arginine kinase Met e 2	1783	74	259
Q3Y8M6.1	*Penaeus aztecus*	Tropomyosin Pen a 1.0102	1069	83	204
ROT66127.1	*Penaeus vannamei*	Actin T2	1323	72	246
XP_038117490.1	*Culex quinquefasciatus*	Arginine kinase Lit v 2	1222	62	217

### Detection of Tc toxin genes

**Fig. 3:**
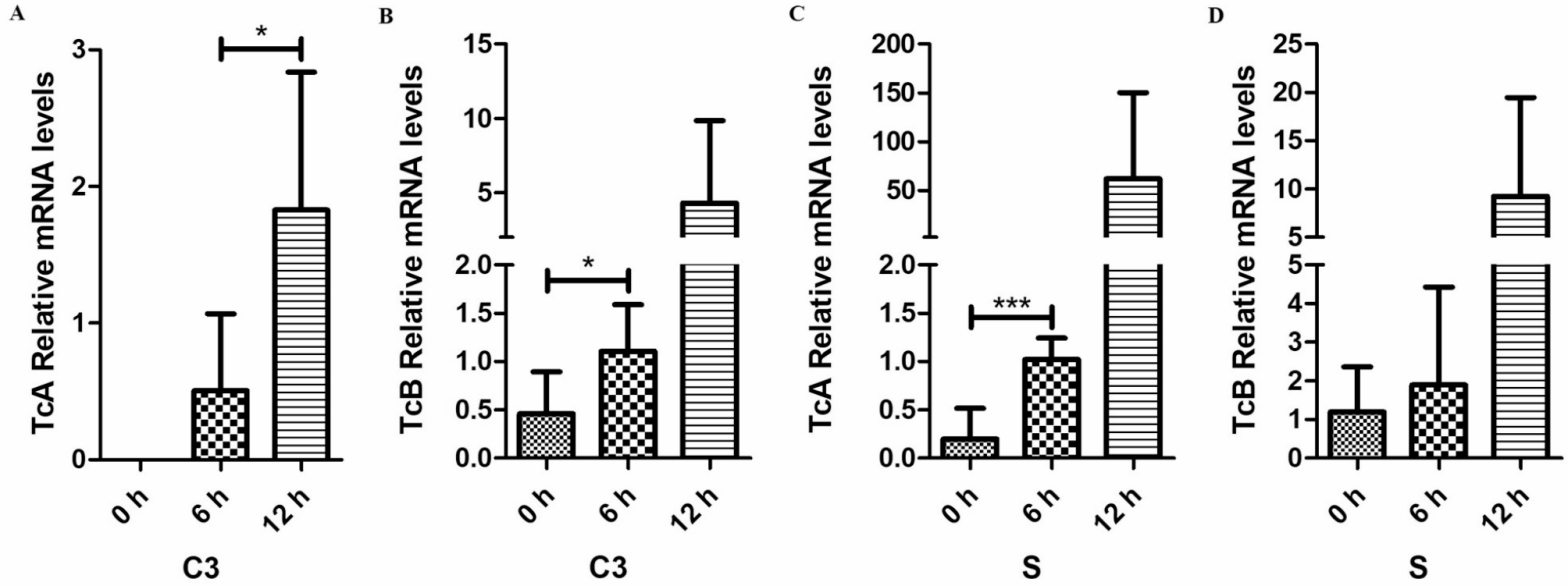
Detection of transcript levels of *Vp*_HLVD_ Tc toxins in shrimp post-larvae. (**A**) Transcript levels of susceptible group C3 TcA subunits. (**B**) Transcript levels of susceptible group C3 TcB subunits. (**C**) Transcript levels of susceptible group S TcA subunits. (**D**) Transcript levels of susceptible group S TcB subunits, with 16S rRNA serving as an internal control. Statistical analysis was performed using a paired Student’s t-test, and asterisks indicate significant differences (*p < 0.05, ***p < 0. 001).

To confirm *Vp*_HLVD_ infection in shrimp, we initially employed qRT-PCR to examine the expression of *Vp*_HLVD_ Tc toxins (TcA and TcB subunits) in individual strain of each species. Our analysis revealed a stark contrast. In the susceptible shrimp group (C3, S), the transcript levels of Tc toxins gradually increased over time as *Vp*_HLVD_ infection progressed (Fig. 3), whereas no Tc toxin transcripts was detected in the disease-resistant shrimp group (B13, B20). This comparison indicated a significantly higher level of *Vp*_HLVD_ in the susceptible shrimp compared to the disease-resistant shrimp. *Vp*_HLVD_ is the main cause of pathogenicity in the susceptible shrimp. This finding further reinforced the resistance advantage of disease-resistant shrimp in combating *Vp*_HLVD_ infections, suggesting the disease-resistant shrimp achieved disease resistance by blocking *Vp*_HLVD_ invasion.

### Transcriptome sequencing analysis and functional annotation

To delve deeper into the molecular mechanisms underlying the disease-resistant response of shrimp against *Vp*_HLVD_, we employed transcriptome sequencing and differential analysis on disease-resistant and susceptible shrimp. We collected three biological replicates from each strain at three distinct time points: the uninfected stage (0 h), the early infection (6 h), the middle and late infection (12 h). In the end, we obtained a total of 36 samples for subsequent transcriptome sequencing analyses.

The RNA samples were sequenced using the Illumina NovaSeq6000 platform, resulting in a total of 222.39 GB of clean data after removing junctions, low-quality sequences, and N-containing reads. At 0, 6, and 12 h, the clean bases were 73.40 GB, 73.70 GB, and 75.29 GB, respectively, with 489,336,760, 491,276,734, and 501,821,168 valid sequences. The data quality was high, with Q20 and Q30 exceeding 96.90% and 92.28%, and a similar GC content (Table S2). The alignment against the reference genome showed an overall efficiency of approximately 86.76%, with 74.47% of reads mapping uniquely. These high-quality data were suitable for further analysis.

**Fig. 4:**
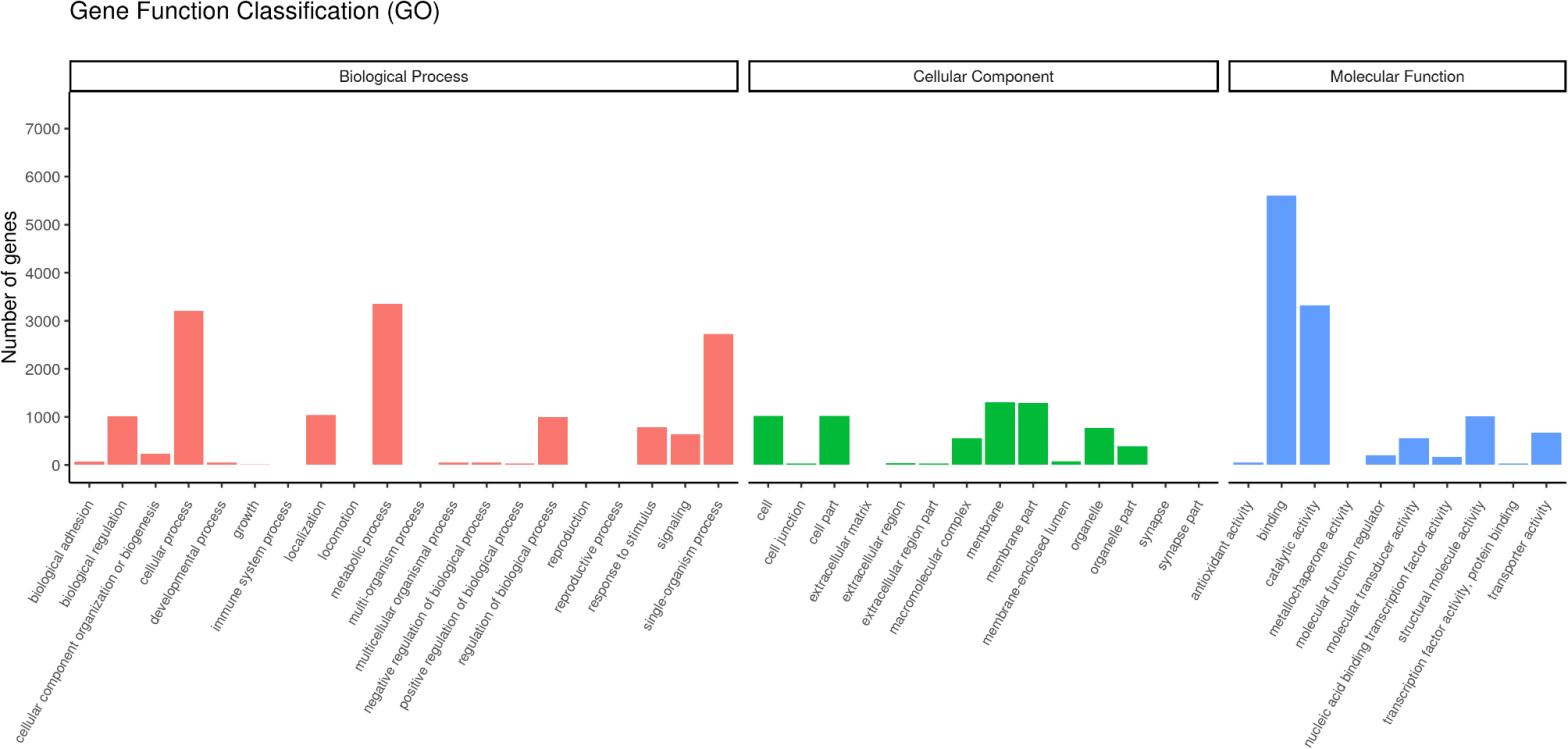
Gene ontology (GO) enrichment analysis.

**Fig. 5:**
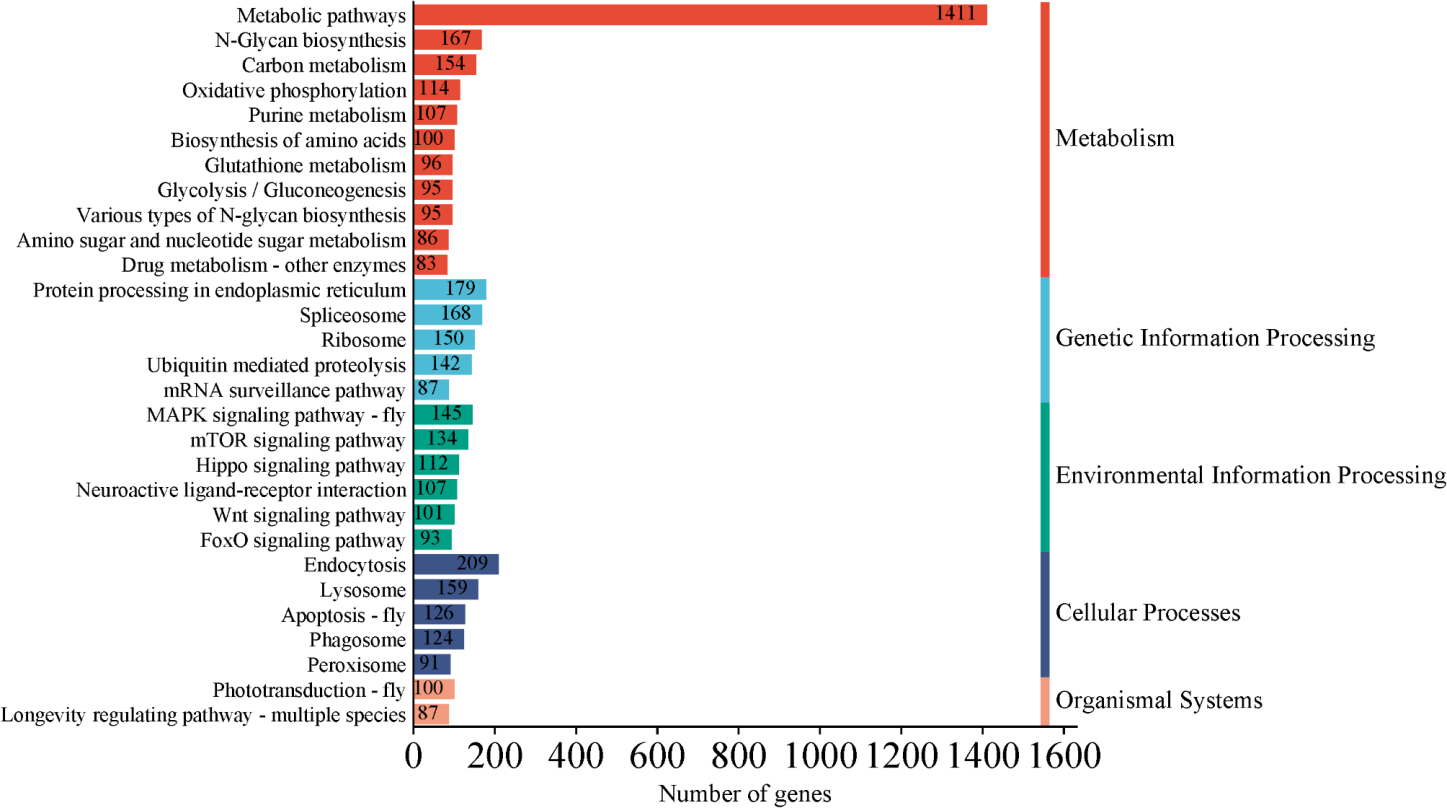
KEGG enrichment analysis.

By splicing the clean reads using Trinity, we obtained 30,768 unigenes, totaling 52,516,693 bp. Individual unigenes varied in length, with the shortest length of 57 bp, the longest length of 40,511 bp, and an average length of about 1707 bp. Further annotation of genes was obtained with a total of 11,311 genes successfully annotated to the GO database (Fig. 4). These genes fell into three major categories: biological process, cellular component, and molecular function. A total of 8244 genes were annotated in the KEGG database (Fig. 5), with enrichment in pathways such as Metabolic pathways, Endocytosis, Protein processing in the endoplasmic reticulum, Spliceosome, N-Glycan biosynthesis, showcasing the diverse biological roles of these genes. This comprehensive annotation provided valuable insights into the functional characteristics of the identified genes.

### DEGs analysis in uninfected shrimp

**Fig. 6:**
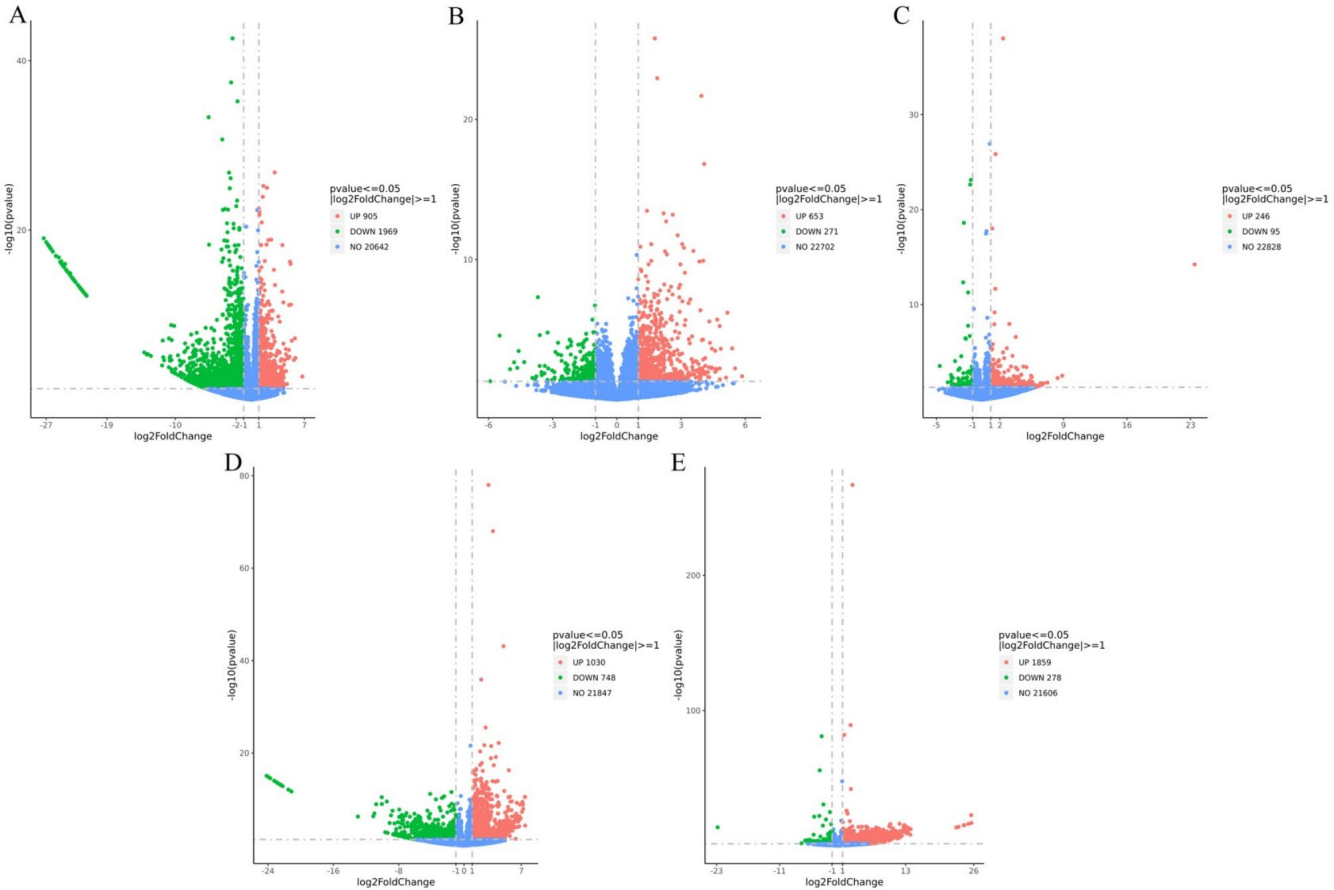
Volcano plots illustrating DEGs in shrimp. (**A**) DEGs in disease-resistant and susceptible shrimp uninfected with *Vp*_HLVD_. (**B**) DEGs in susceptible shrimp at 6 h post-infection compared to 0 h. (**C**) DEGs in disease-resistant shrimp at 6 h post-infection compared to 0 h. (**D**) DEGs in susceptible shrimp at 12 h post-infection compared to 0 h. (**E**) DEGs in disease-resistant shrimp at 12 h post-infection compared to 0 h.

To investigate gene expression differences, we employed the DESeq2 statistical approach, adopting a fold change threshold of |log2(fold change)|≥ 1 and an adjusted *p*-value < 0.05 as the criteria for significant differential expression. Initially, we compared gene expression between disease-resistant shrimp and susceptible shrimp to identify differential patterns in resistance. In uninfected shrimp (0 h), disease-resistant individuals exhibited significant differential gene expression, with 2874 genes differentially expressed, including 905 up-regulated and 1969 down-regulated genes compared to the susceptible shrimp group (Fig. 6A). Among the up-regulated genes, we found that 35 were linked to *Vibrio* immunity, such as serine protease inhibitors, lysozyme-like proteins, C-type lectin domain family 7 member A-like, perlucin-like, and peroxidase-like genes. Conversely, 39 down-regulated genes were involved in *Vibrio* infections, including five toxin-binding genes (aminopeptidase N-like, methionine aminopeptidase 1-like, cyclin-dependent kinase 1-like, elongation factor 2-like, and filamin-A-like), as well as two genes promoting *Vibrio* colonization (N-acetylneuraminate lyase-like). These data showed that the gene expression dynamics between susceptible and disease-resistant shrimp revealed distinct patterns, with disease-resistant shrimp displaying an advantage in immunity (Table 2).

**Table 2:** DEGs associated with *Vibrio* immunity or infections in two shrimp species (susceptible and disease-resistant) when uninfected with *Vp*_HLVD_.

Gene name	log2FoldChange (A0 h vs I0 h)	Regulated	Gene description
LOC113814570	1.592468152	Up	serine protease inhibitor I/II-like
LOC113819745	1.779476241	Up	hemolymph clottable protein-like
LOC113826535	1.03245363	Up	serine/threonine-protein kinase pim-1-like
LOC113815655	1.247379258	Up	ladderlectin-like
LOC113822590	2.983397783	Up	Kruppel homolog 1-like
LOC113826536	1.250313874	Up	serine/threonine-protein kinase pim-3-like
LOC113828680	1.764189069	Up	CLIP domain-containing serine protease 2-like
LOC113800674	3.975080234	Up	WAP four-disulfide core domain protein 2-like
LOC113816158	1.011766577	Up	galactoside 2-alpha-L-fucosyltransferase 3-like
LOC113819139	3.873091998	Up	fatty acid binding protein 1-B.1-like
LOC113815775	1.377403027	Up	WAP four-disulfide core domain protein 5-like
LOC113810339	1.399937273	Up	TNF receptor-associated factor 6-like
LOC113806222	1.228644267	Up	ficolin-1-like
LOC113805933	4.582256254	Up	lysozyme-like
LOC113812696	2.421947933	Up	leucine-rich repeat protein SHOC-2-like
LOC113812977	3.40478086	Up	C-type lectin domain family 7 member A-like
LOC113825293	2.964314327	Up	heat shock protein 67B1-like
LOC113813554	1.189513483	Up	ficolin-1-like
LOC113825262	2.509210642	Up	heat shock protein 27-like
LOC113800863	3.386909259	Up	laccase-like
LOC113801859	2.017421772	Up	heat shock protein 67B1-like
LOC113825012	3.065033591	Up	fatty acid binding protein 1-B.1-like
LOC113824920	2.698263903	Up	perlucin-like protein
LOC113816541	2.041294572	Up	UNC93-like protein
LOC113828228	2.250146831	Up	carbohydrate sulfotransferase 10-like
LOC113806298	2.054336559	Up	serine protease inhibitor 42Dd-like
LOC113826335	2.29382525	Up	carbohydrate sulfotransferase 3-like
LOC113807934	2.74879397	Up	peroxidase-like
LOC113826359	3.165371047	Up	glutathione peroxidase-like
LOC113801332	2.094812965	Up	galactoside 2-alpha-L-fucosyltransferase 1-like
LOC113815763	1.804982647	Up	crustacean hyperglycemic hormone-like
LOC113827379	1.405413673	Up	granulins-like
LOC113824082	1.019295491	Up	protein ERGIC-53-like
LOC113807930	2.60341283	Up	peroxidase-like
LOC113802306	1.43585118	Up	lysozyme C-like
LOC113814070	− 3.382499528	Down	methionine aminopeptidase 1-like
LOC113804788	− 1.545126398	Down	aminopeptidase N-like
LOC113828411	− 1.136844238	Down	filamin-A-like
LOC113804808	− 2.916618036	Down	N-acetylneuraminate lyase-like
LOC113818305	− 2.263248658	Down	cyclin-dependent kinase 1-like
LOC113818954	− 3.068396081	Down	N-acetylneuraminate lyase-like
LOC113811541	− 1.380517999	Down	elongation factor 2-like
LOC113806043	− 1.310867168	Down	heat shock protein HSP 90-alpha
LOC113808481	− 2.70415034	Down	C-type lectin-like
LOC113813073	− 1.159174576	Down	C-type lectin domain family 6 member A-like
LOC113828953	− 1.216690409	Down	elongation of very long chain fatty acids protein 4-like
LOC113816031	− 1.213635558	Down	heat shock protein 60A-like
LOC113820679	− 2.789827349	Down	heat shock protein 22-like
LOC113816317	− 1.808256361	Down	troponin C-like
LOC113829109	− 1.641306774	Down	histone H2A-like
LOC113812874	− 5.750815558	Down	alkaline phosphatase-like
LOC113817521	− 3.785304917	Down	alkaline phosphatase-like
LOC113817212	− 3.276587727	Down	alkaline phosphatase-like
LOC113812869	− 4.732941843	Down	alkaline phosphatase-like
LOC113815640	− 2.00047406	Down	heat shock 70 kDa protein cognate 4-like
LOC113828795	− 8.020477671	Down	peroxidase-like
LOC113819893	− 1.581598874	Down	glutathione peroxidase 2-like
LOC113828778	− 7.778978505	Down	peroxidase-like protein 2
LOC113807222	− 1.178251441	Down	beta-1,3-glucan-binding protein
LOC113822223	− 5.716455639	Down	peritrophin-1-like
LOC113822219	− 5.149112898	Down	peritrophin-1-like
LOC113822213	− 4.427611425	Down	peritrophin-1-like
LOC113822214	− 4.277847413	Down	peritrophin-1-like
LOC113822217	− 4.340133664	Down	peritrophin-1-like
LOC113818031	− 1.787532421	Down	ctenidin-1-like
LOC113809720	− 1.200693981	Down	leucine-rich repeat protein 1-like
LOC113822052	− 1.522455681	Down	astakine
LOC113803303	− 1.032021394	Down	macrophage migration Inhibitory factor-like
LOC113819742	− 1.053496245	Down	hemolymph clottable protein-like
LOC113808543	− 1.891272065	Down	chelonianin-like
LOC113815071	− 1.154325927	Down	superoxide dismutase [Cu–Zn]-like
LOC113806235	− 1.0900778	Down	WAP four-disulfide core domain protein 18-like
LOC113805224	− 6.211561913	Down	perlucin-like
LOC113822897	− 2.604689917	Down	lysM and putative peptidoglycan-binding domain-containing protein 2-like

**Fig. 7:**
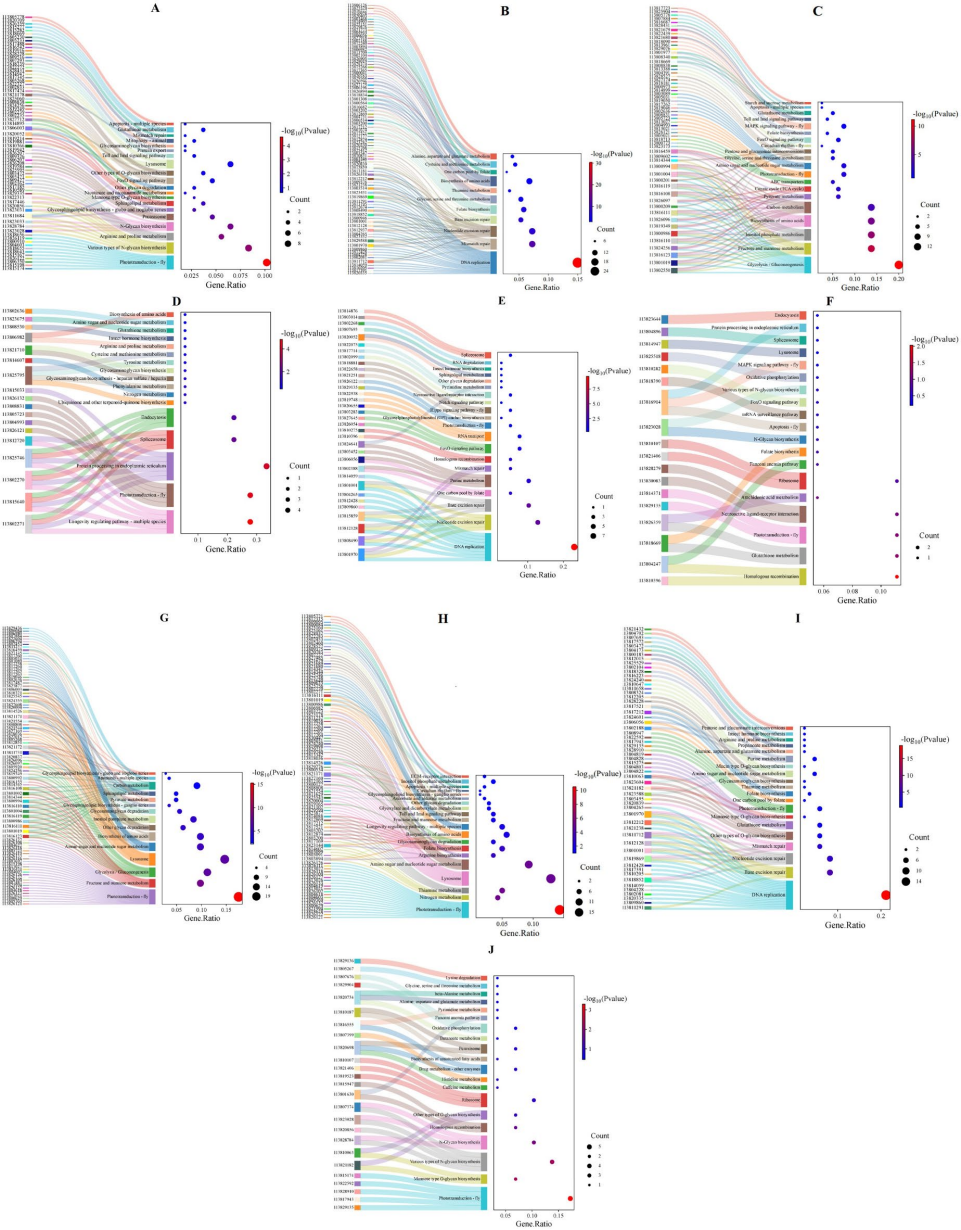
Sankey diagrams illustrating KEGG pathway enrichment for DEGs in shrimp^76–78^. (**A**) Upregulated DEGs in uninfected *Vp*_HLVD_-resistant shrimp. (**B**) Downregulated DEGs in uninfected *Vp*_HLVD_-resistant shrimp, using uninfected susceptible shrimp as a control. (**C**) Upregulated DEGs in susceptible shrimp at 6 h post-infection with *Vp*_HLVD_. (**D**) Upregulated DEGs in disease-resistant shrimp at 6 h post-infection. (**E**) Downregulated DEGs in susceptible shrimp at 6 h post-infection. (**F**) Downregulated DEGs in disease-resistant shrimp at 6 h post-infection. (**G**) Upregulated DEGs in susceptible shrimp at 12 h post-infection. (**H**) Upregulated DEGs in disease-resistant shrimp at 12 h post-infection. (**I**) Downregulated DEGs in susceptible shrimp at 12 h post-infection. (**J**) Downregulated DEGs in disease-resistant shrimp at 12 h post-infection.

**Fig. 8:**
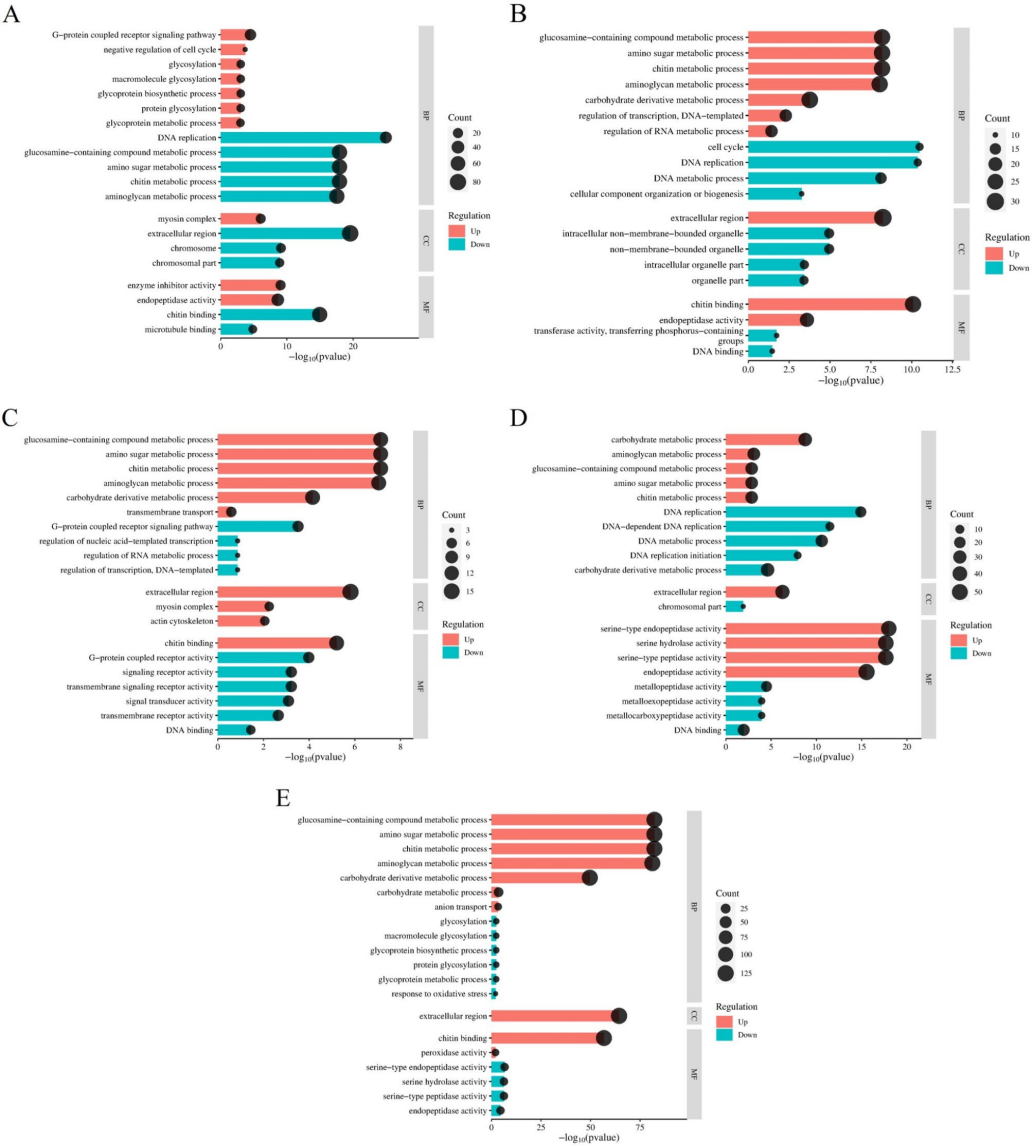
Line graphs illustrating GO annotations for DEGs in shrimp. (**A**) GO annotations for DEGs in disease-resistant and susceptible shrimp uninfected with *Vp*_HLVD_. (**B**) GO annotations for DEGs in susceptible shrimp at 6 h post-infection with *Vp*_HLVD_. (**C**) GO annotations for DEGs in disease-resistant shrimp at 6 h post-infection. (**D**) GO annotations for DEGs in susceptible shrimp at 12 h post-infection. (**E**) GO annotations for DEGs in disease-resistant shrimp at 12 h post-infection.

The KEGG analysis of these DEGs indicated that the up-regulated genes in disease-resistant shrimp were predominantly enriched in pathways such as phototransduction, N-glycosylation biosynthesis, and amino acid metabolism, with a notable emphasis on immune-related pathways like the FoxO signaling pathway, lysosome, and Toll/IMD signaling pathway, Apoptosis (Fig. 7A). Conversely, the down-regulated genes were primarily concentrated in DNA replication and repair processes, alongside amino acid metabolism pathways, and showed a reduced involvement in immune-related pathways (Fig. 7B). GO analysis revealed that the up-regulated genes were mainly engaged in activities like enzyme inhibitor activity and endopeptidase activity, whereas the down-regulated genes were primarily focused on biological and molecular processes such as DNA replication, extracellular regions, and chitin binding (Fig. 8A). These findings suggested that disease-resistant shrimp exhibit a heightened baseline expression of immune-related genes, which bolsters their ability to combat *Vp*_HLVD_ infections.

### DEGs analysis in ***Vp***_HLVD_-infected shrimp

Subsequently, we took shrimp infected with *Vp*_HLVD_ for 6 and 12 h as the experimental group, contrasting them with uninfected controls (0 h) to analyze temporal gene expression changes during infection. Upon exposure to *Vp*_HLVD_, a stark contrast in gene expression was observed between susceptible and disease-resistant shrimp. Six hours post-infection, susceptible shrimp exhibited significant alterations in 924 genes, with 653 up-regulated and 271 down-regulated (Fig. 6B), indicating a more extensive response. In contrast, disease-resistant shrimp showed a milder response with only 341 genes affected, including 246 up-regulated and 95 down-regulated genes (Fig. 6C). The overall gene changes were more numerous in the susceptible shrimp, with a higher number of up-regulated genes compared to down-regulated ones. This suggested that both shrimp populations responded to the infection, but the susceptible shrimp displayed a more pronounced early-stage response to *Vp*_HLVD_.

Following differential gene analysis, we performed KEGG pathway enrichment analysis to uncover potential biological pathways, while GO analysis was employed to delve deeper into the specific cellular functions and molecular processes associated with these genes. We conducted comparative GO analysis of the up- and down-regulated DEGs in resistant and susceptible shrimp at various time points post-infection. KEGG analysis revealed that the up-regulated genes in susceptible shrimp were primarily involved in glycolysis/gluconeogenesis, fructose and mannose metabolism, and Inositol phosphate signaling, with immune pathways such as FoxO signaling, MAPK signaling, Toll/IMD pathways, glutathione metabolism, and apoptosis standing out (Fig. 7C). In contrast, disease-resistant shrimp exhibited up-regulation in genes linked to longevity regulation, phototransduction, protein processing in endoplasmic reticulum, and spliceosome, with immune-related pathways like endocytosis being more prominent (Fig. 7D). The down-regulated genes in susceptible shrimp were predominantly focused on DNA replication, nucleotide excision repair, and base excision repair (Fig. 7E), whereas in disease-resistant shrimp down-regulated genes were centered on homologous recombination, glutathione metabolism, and phototransduction (Fig. 7F). These findings underscored the distinct differences in gene response and activation of immune pathways between susceptible and disease-resistant shrimp when confronted with *Vp*_HLVD_ infection.

In the GO analysis, the up-regulated genes in susceptible shrimp were primarily associated with chitin metabolism, aminoglycan metabolism, and extracellular region composition, with functions like chitin binding and endopeptidase activity. Conversely, down-regulated genes were mainly linked to cellular processes like cell cycle and DNA replication, as well as cellular components such as intracellular non-membrane-bound organelles (Fig. 8B). In contrast, disease-resistant shrimp exhibited up-regulation in chitin and amino sugar metabolism within the extracellular region, while their down-regulated genes were predominantly involved in G protein-coupled receptor signaling and transmembrane receptor activity (Fig. 8C). Notably, both populations displayed a similar pattern of up-regulated gene concentration at the 6-h post-infection time point.

At the 12-h post-infection with *Vp*_HLVD_, a substantial difference in gene expression was observed between susceptible and disease-resistant shrimp. In susceptible shrimp, 1778 genes were significantly altered, with 1030 up-regulated and 748 down-regulated (Fig. 6D), indicating a more extensive response. Conversely, disease-resistant shrimp exhibited a more robust gene response with 2137 genes affected, of which 1859 were up-regulated and 278 down-regulated (Fig. 6E). The total gene changes and up-regulated genes were higher in disease-resistant shrimp, while susceptible shrimp had a higher number of down-regulated genes. This suggested that in the later stages of infection, the defense strategy of disease-resistant shrimp diverged from that of susceptible shrimp, with the peak of gene response occurring later in the disease-resistant shrimp.

The KEGG analysis revealed that up-regulated genes in susceptible shrimp were primarily concentrated in phototransduction, fructose and mannose metabolism, and glycolysis/gluconeogenesis, with immune pathways like lysosome and apoptosis standing out (Fig. 7G). In contrast, disease-resistant shrimp exhibited up-regulation in genes related to phototransduction, nitrogen metabolism, and thiamine metabolism, displaying a stronger immune response through pathways such as lysosome, Toll/IMD signaling, apoptosis, phagolysosomes, and the MAPK signaling pathway (Fig. 7H). After 12 h of infection, disease-resistant shrimp showed a significantly higher number of up-regulated immune-related pathways compared to susceptible shrimp, indicating heightened immunological activity in the intermediate and later stages of *Vp*_HLVD_ infection. Furthermore, the down-regulated genes in susceptible shrimp primarily focused on DNA replication, base excision repair, and nucleotide excision repair (Fig. 7I), whereas disease-resistant shrimp’s down-regulated genes centered on phototransduction, mannose-type O-glycan biosynthesis, and N-glycan biosynthesis (Fig. 7J). Notably, the genes significantly down-regulated in susceptible shrimp at both 6 and 12 h post-infection were predominantly enriched in DNA damage repair pathways, suggesting that these pathways in susceptible shrimp were strongly affected by *Vp*_HLVD_ infection, which may hinder the susceptible shrimp’s ability to resist *Vp*_HLVD_ invasion.

The GO analysis demonstrated that up-regulated genes in susceptible shrimp primarily contributed to endopeptidase activity and serine endopeptidase function, while down-regulated genes were centered on biological processes like carbohydrate derivative metabolism (Fig. 8D). In contrast, disease-resistant shrimp’s up-regulated genes were concentrated on molecular functions such as chitin metabolism, aminoglycan metabolism, extracellular regions, and chitin binding, indicating a distinct immune response. The down-regulated genes in disease-resistant shrimp resembled those up-regulated in susceptible shrimp, particularly in endopeptidase-related molecular functions (Fig. 8E), suggesting a different mechanism in *Vp*_HLVD_ infection response.

### Venn analysis in susceptible shrimp upon to ***Vp***_HLVD_

To further investigate the genes of shrimp involved in *Vp*_HLVD_ infection, we performed gene expression profiling on susceptible shrimp. The analysis revealed that 412 genes were up-regulated at both 6 and 12 h post-infection compared to the uninfected control. Among these, 301 genes displayed consistent up-regulation, with their expression peaking at 12 h (log2FoldChange from 6 to 0 h was lower than that from 12 to 0 h).

Of the 301 genes examined for sustained upregulation, a significant concentration was observed in key immune pathways, notably the FoxO pathway, lysosome pathway, Notch pathway, Apoptosis pathway, Phagosome pathway, Toll/IMD pathway, MAPK pathway, Hippo pathway, and Wnt pathway. Additionally, genes which contribute to antimicrobial peptide production (like anti-lipopolysaccharide factor-like, ctenidin-1-like, and apolipoprotein D-like), lectin regulation (perlucin-like proteins and C-type lectin family 6 member A-like), transferrin transport (transferrin-like), and chitinase defense (chitinase-3-like protein 1 and acidic mammalian chitinase-like) were also found (Table 3). Further analysis of these genes in disease-resistant shrimp revealed that lysozyme-like, C-type lectin domain family 7 member A-like, apolipoprotein D-like, perlucin-like protein, leucine-rich repeat protein SHOC-2-like, CREB-binding protein-like 2C transcript variant X1, low-density lipoprotein receptor-like, phosphoenolpyruvate carboxykinase 2C cytosolic [GTP]-like, and phenoloxidase-activating factor 3-like genes had significantly higher baseline expression levels compared to susceptible shrimp. This indicated their key roles in resisting *Vibrio* infection.

**Table 3:** DEGs involved in *Vibrio* infection in susceptible shrimp.

Gene name	log2FoldChange(I6 h vs I0 h)	log2FoldChange(I12 h vs I0 h)	Regulated	Gene description
LOC113805933	5.39	7.48	Up	lysozyme-like
LOC113817262	3.80	6.75	Up	acidic mammalian chitinase-like
LOC113812851	3.71	5.82	Up	sphingomyelin phosphodiesterase-like 2C transcript variant X1
LOC113812977	3.67	7.21	Up	C-type lectin domain family 7 member A-like
LOC113819046	3.58	4.24	Up	chitinase-3-like protein 1
LOC113810108	3.20	4.29	Up	anti-lipopolysaccharide factor-like
LOC113815027	3.05	6.45	Up	apolipoprotein D-like
LOC113805418	2.96	6.20	Up	lysozyme-like
LOC113808808	2.94	5.09	Up	cathepsin L1-like
LOC113822012	2.90	4.25	Up	–
LOC113812976	2.75	5.49	Up	C-type lectin domain family 6 member A-like
LOC113824920	2.72	5.82	Up	perlucin-like protein
LOC113813021	2.34	2.73	Up	actin, cytoplasmic A3a-like
LOC113820005	2.04	4.27	Up	perlucin-like protein
LOC113823275	2.02	2.53	Up	phenoloxidase-activating factor 1-like
LOC113807950	1.97	3.20	Up	transferrin-like
LOC113808838	1.85	2.54	Up	CREB-binding protein-like 2C transcript variant X1
LOC113807955	1.78	2.84	Up	transferrin-like
LOC113810940	1.78	2.42	Up	low-density lipoprotein receptor-like
LOC113818090	1.70	1.74	Up	pollen-specific leucine-rich repeat extensin-like protein 2
LOC113827169	1.70	3.19	Up	lysosomal aspartic protease-like
LOC113800363	1.64	2.29	Up	anti-lipopolysaccharide factor-like
LOC113820510	1.56	2.78	Up	anti-lipopolysaccharide factor-like
LOC113819349	1.48	1.97	Up	phosphoenolpyruvate carboxykinase, 2C cytosolic [GTP]-like
LOC113809599	1.46	1.33	Up	–
LOC113828431	1.38	2.94	Up	phenoloxidase-activating factor 3-like
LOC113807884	1.27	1.51	Up	baculoviral IAP repeat-containing protein 7-B-like
LOC113826097	1.22	1.74	Up	phosphoenolpyruvate carboxykinase, 2C cytosolic [GTP]-like
LOC113808882	1.14	1.22	Up	putative fatty acyl-CoA reductase CG5065
LOC113812696	1.12	1.35	Up	leucine-rich repeat protein SHOC-2-like
LOC113805243	1.12	3.78	Up	perlucin-like protein
LOC113813961	1.09	1.17	Up	tyrosine-protein kinase SRK2-like
LOC113801835	1.04	1.22	Up	ctenidin-1-like

### Validation of DEGs

To validate the reliability of the analyzed results, we randomly selected 11 DEGs for qRT-PCR verification. As depicted in Table 4, genes consistently up-regulated in susceptible shrimp during *Vibrio* exposure included anti-lipopolysaccharide factor-like, ctenidin-1-like, C-type lectin domain family 6 member A-like, perlucin-like protein, leucine-rich repeat protein SHOC-2-like, and lysozyme-like, among others. Meanwhile, three genes—C-type lectin domain family 7 member A-like, peroxidase-like, and glutathione peroxidase-like—displayed differential expression between susceptible and disease-resistant shrimp at the uninfected stage (Table 4). The qRT-PCR results demonstrated a high degree of consistency with transcriptome data, reinforcing the reliability of transcriptome sequencing analysis.

**Table 4:** Validation of DEGs.

Gene name	Gene description	RNA-seq			qRT-PCR(2^-ΔΔCt^)		
Validation of DEGs that are consistently upregulated in susceptible shrimp after *Vp*_HLVD_ infection							
		0 h	6 h	12 h	0 h	6 h	12 h
LOC113817212	anti-lipopolysaccharide factor-like	490.84 ± 265.78	1526.60 ± 374.80	13,260.95 ± 1807.56	1.19 ± 0.58	19.04 ± 5.76	45.02 ± 9.02
LOC113810108	anti-lipopolysaccharide factor-like	674.036 ± 763.25	6203.228 ± 4158.75	13,260.95 ± 10,628.21	1.00 ± 0.09	33.22 ± 1.03	102.02 ± 13.11
LOC113812976	C-type lectin domain family 6 member A-like	2.17 ± 4.42	14.54 ± 15.24	98.11 ± 106.06	1.06 ± 0.36	1.65 ± 0.67	88.47 ± 10.38
LOC113812696	leucine-rich repeat protein SHOC-2-like	817.34 ± 451.91	1772.04 ± 484.36	2097.21 ± 1020.76	1.01 ± 0.12	1.04 ± 0.10	1.86 ± 0.04
LOC113805933	lysozyme-like	0	7.59 ± 11.22	32.52 ± 28.84	1.04 ± 0.29	2.39 ± 0.38	78.05 ± 7.36
LOC113805418	lysozyme-like	0.17 ± 0.37	1.97 ± 1.15	18.76 ± 18.60	1.11 ± 0.51	5.81 ± 1.06	183.40 ± 20.38
LOC113824920	perlucin-like protein	5.81 ± 11.33	38.36 ± 32.92	330.13 ± 351.98	1.04 ± 0.28	14.21 ± 3.94	1605.69 ± 90.82
LOC113801835	ctenidin-1-like	898.97 ± 698.31	1852.01 ± 319.07	2107.90 ± 1415.55	1.00 ± 0.08	1.89 ± 0.19	14.85 ± 0.78

Comparison of gene expression levels between susceptible and disease-resistant shrimp groups at the uninfected stage							
		A 0 h	I 0 h		A 0 h	I 0 h	
LOC113812977	C-type lectin domain family 7 member A-like	7.28 ± 8.38	0.66 ± 0.77		2.69 ± 1.22	0.78 ± 0.80	
LOC113826359	glutathione peroxidase-like	1.69 ± 1.12	0		10.67 ± 2.67	0.77 ± 0.23	
LOC113807934	peroxidase-like	2.55 ± 2.09	0.31 ± 0.68		7.46 ± 0.52	1.58 ± 0.73	

I: susceptible shrimp; A: disease-resistant shrimp.

The data in the table are ‘mean ± standard deviation’.

## Discussion

The etiological agent responsible for HLVD has been identified as virulent strains of *V. parahaemolyticus* (*Vp*_HLVD_). To combat this disease effectively and prevent future outbreaks, selective breeding programs for shrimp have been implemented, leading to the development of a new strain (disease-resistant shrimp) with enhanced resistance to HLVD. However, further research is necessary to unravel the underlying mechanisms that confer this improved disease resistance.

This study employed artificial infection to confirm the disease resistance of shrimp populations to *Vp*_HLVD_. Susceptible shrimp exhibited high mortality rates and typical pathologies, reinforcing the identification of *Vp*_HLVD_ as the primary causative agent. In contrast, disease-resistant shrimp displayed significantly lower mortality rates and maintained good health upon infection, demonstrating their effective defense mechanisms against HLVD. The comparison between susceptible and resistant populations highlighted the varying levels of susceptibility and the importance of immune response in combating this disease.

Fecal analysis is a valuable tool for disease surveillance in shrimp aquaculture, as it provides insights into the health status of shrimp. For instance, White Feces Syndrome (WFS) was characterized by white, stringy, foul-smelling feces that float on the water surface^31,32^. In our study, we observed fecal adhesion in susceptible shrimp infected with *Vp*_HLVD_, indicating a disruption of their digestive system. Through LC–MS/MS analysis of the fecal matter, we identified cytoskeletal proteins, such as Myosin heavy chain type 2, Fast-type skeletal muscle actin 15, Actin T2, and Tropomyosin Pen a 1.0102, as primary components. These proteins, integral components of the contractile machinery in muscle tissue^33^, hinted at potential muscular breakdown within the shrimp’s digestive tract. The presence of these proteins in feces could be indicative of *Vp*_HLVD_-induced proteolytic activity or the release of toxins that target cytoskeletal structures. Previous research has established a link between *Vibrio* toxins and their ability to directly or indirectly target cytoskeletal proteins^34,35^. For instance, the VopV effector protein produced by *V. parahaemolyticus* binds to F-actin, leading to its accumulation and detrimental effects on intestinal function^34^. Additionally, the VopF protein from *Vibrio cholerae* interacted with actin to form actin-rich filaments, promoting cell protrusions that facilitate bacterial colonization^36,37^. Recently, a study identified Tc toxins produced by *Vp*_HLVD_, which contribute significantly to the bacterium’s pathogenicity^10^. These toxins were capable of inducing HLVD in post-larvae shrimp. Thus, Tc toxins produced by *Vp*_HLVD_ might target cytoskeletal proteins, either directly or indirectly, leading to impaired cell function and the observed digestive system disruption in susceptible shrimp.

The discovery that Tc toxins are the primary causative agents of disease in susceptible shrimp naturally leads to the question: Do disease-resistant shrimp combat the disease by preventing the toxins from exerting their harmful effects? The expression of Tc toxin subunits is unique to the *Vp*_HLVD_ strain^10^, which has prompted the proposal of analyzing transcript levels of Tc toxins as a diagnostic tool for detecting *Vp*_HLVD_ infection in shrimp. Interestingly, our study revealed contrasting trends in susceptible and disease-resistant shrimp groups. As *Vibrio* infection progressed, the susceptible group showed a gradual increase in Tc toxin (TcA and TcB) transcript levels, indicating the shrimp were affected by *Vp*_HLVD_ infection. Conversely, no Tc toxin transcripts was detected in the disease-resistant group, suggesting their ability to effectively either clear the bacteria or prevent *Vp*_HLVD_ invasion.

To elucidate the disease resistance mechanism in shrimp, we first conducted a comprehensive analysis of DEGs in *Vp*_HLVD_-uninfected susceptible shrimp and disease-resistant shrimp. Our findings suggested that disease-resistant shrimp exhibited enhanced defense mechanisms against *Vp*_HLVD_ compared to their susceptible counterparts. A key observation was the higher involvement of immune-related genes in disease-resistant shrimp, widely reported in previous studies related to *Vibrio* immunity, including serine protease inhibitor I/II-like, Kruppel homolog 1-like, ficolin-1-like, and granulins-like. Research had demonstrated the significant role of serine protease inhibitors in immune responses. For example, serine protease inhibitor 1 enhances the clearance of *V. anguillarum* in *Marsupenaeus japonicus*^38^, and a Kazal-type serine protease inhibitor from *Penaeus monodon* inhibits the growth of various *Vibrio* species^39^. Kruppel homolog 1-like is crucial for regulating the response to *V. parahaemolyticus* (AHPND) infection in *P. vannamei* by modulating antioxidant enzymes and facilitating the nuclear entry of Relish^40^. Furthermore, ficolin, vital for invertebrate innate immunity, protects hepatopancreatic cells of *Procambarus clarkii* from *V. parahaemolyticus*^41^ and enhances bacterial clearance and phagocytosis in *Macrobrachium nipponense* and *Portunus trituberculatus*^42,43^. Granulins-like peptides exhibited potent bactericidal activity against *Vibrio* species, including *V. vulnificus*, *V. alginolyticus*, and *V. harveyi*^44^.

Furthermore, our analysis revealed that the highly expressed genes in disease-resistant shrimp were more enriched in immune-related pathways, such as the Toll/IMD signaling pathway, apoptosis, and FoxO signaling pathway, Lysosome. This suggested a more active immune response in these shrimps. The Toll/IMD signaling pathway is crucial for immune gene expression in shrimp, especially against bacterial and WSSV infections. It is activated in *P. vannamei* during *V. anguillarum* infestation^45–47^. Apoptosis is vital for controlling *Vibrio* invasions, contributing to homeostasis against *V. harveyi* in *Macrobrachium rosenbergii*^21^, and is significant in the *V. parahaemolyticus* immune response of *P. vannamei*^48^. The FoxO pathway enhances immune defense by promoting antimicrobial peptide expression and aiding pathogen phagocytosis through the IMD pathway^49^. The Lysosome pathway is essential for digesting extracellular substances and killing pathogens via phagocytosis. Its role in shrimp immunity against *Vibrio* is supported by transcriptome analyses of *P. vannamei* and *Neocaridina denticulata sinensis*^50–53^. Additionally, the importance of lysosome and phagosome pathways is highlighted in *Vibrio coralolyticus*-infected *Mesocentrotus nudus*^54^. Therefore, our findings indicated that disease-resistant shrimp mount a more robust immune response to *Vp*_HLVD_ by up-regulating key immune-related genes and pathways.

Our analysis showed that susceptible shrimp express higher levels of genes that bind to *Vibrio* toxins compared to disease-resistant shrimp. Genes such as aminopeptidase N-like, cyclin-dependent kinase 1-like, elongation factor 2-like, filamin-A-like, and actin were upregulated in susceptible shrimp. The role of aminopeptidase N family proteins, LvAPN1 and LvAPN2, as receptors for insect cry toxins has been established. Notably, LvAPN1 in *P. vannamei* facilitated the entry of the *Vp*_AHPND_ toxin produced by *V. parahaemolyticus* into hemocytes, making it a critical factor in toxin-host interactions^55^. The MARTX toxin RRSP from *V. vulnificus* binds to cyclin-dependent kinase 1, affecting mitotic progression^56^, and the cholix toxin from *V. cholerae* inhibits host protein synthesis by targeting elongation factor 2^57–59^. Filamin-A aids in *Vibrio* pathogenesis by mediating bacterial toxin effects, such as *V. vulnificus* RtxA1 toxin-induced cytoskeletal rearrangement^60^, and T3SS2 effector VopV from *V. parahaemolyticus* interacts with filamin and actin^61^. Interestingly, we also found that genes promoting *Vibrio* colonization, like N-acetylneuraminate lyase-like (NanA), were upregulated in susceptible shrimp. NanA is upregulated in susceptible shrimp and aids *Vibrio* colonization^62,63^. The lower expression of these genes in disease-resistant shrimp suggests fewer *Vibrio* toxin-binding receptors and colonization-promoting genes, contributing to their resilience against HLVD. These findings highlight the superior defenses of disease-resistant shrimp against *Vibrio* invasion.

At 6 h post-infection with *Vp*_HLVD_, susceptible shrimp displayed a stronger initial gene expression response, with active immune pathways like FoxO, Toll and IMD, and Apoptosis, similar to those in uninfected disease-resistant shrimp. This suggests they may attempt to utilize similar immune strategies. By 12 h, disease-resistant shrimp showed a heightened genetic response, further activating pathways such as Lysosome, Apoptosis and Toll and IMD. Conversely, in susceptible shrimp, down-regulated genes at both 6 and 12 h highlighted diminished defenses, with enriched pathways in DNA damage repair—DNA replication, Base excision repair, and Nucleotide excision repair—indicating reduced capability to repair damage similar to *V. alginolyticus* infections in *P. vannamei* hemocytes^64^. This down-regulation suggests their limited resistance to *Vibrio* invasion compared to disease-resistant shrimp.

GO analysis revealed that up-regulated DEGs were enriched in chitin metabolic processes, amino sugar metabolic processes and the extracellular region in both susceptible shrimp at 6 h and disease-resistant shrimp at 6 and 12 h post-infection with *Vp*_HLVD_. Chitin serves as a nutrient source for *Vibrio*, aiding in biofilm formation and competence^65–67^. Increased expression of chitin catabolism genes, including chitotriosidase-1-like and chitinase-3-like protein, suggests this process might reduce *Vibrio* adherence and enhance host defenses. Amino sugar metabolism, enriched in stressed shrimp, is crucial for resilience and could aid in defense against *Vp*_HLVD_^68,69^. Interestingly, serine-type endopeptidase-related genes were up-regulated in susceptible shrimp and down-regulated in disease-resistant shrimp at 12 h. Serine proteases, *Vibrio* virulence factors, exhibit cytotoxic effects and support host invasion^70,71^. *V. cholerae* proteases modify host lectins, reducing binding to *Vibrio*^72^, while other pathogens exploit host proteases for disease progression^73,74^. *Vp*_HLVD_ might exploit host serine proteases similarly, whereas disease-resistant shrimp may counteract these effects through gene down-regulation.

In order to investigate *Vibrio*’s effects on shrimp, we further identified 301 genes with consistently elevated expression levels in susceptible shrimp, involving metabolic and immune processes like fructose and mannose metabolism, carbon metabolism, transferrin transport, and the citrate cycle. Key genes highly expressed include antimicrobial peptide-related genes, such as anti-lipopolysaccharide factor-like (ALF) and ctenidin-1-like, as well as lectin-related genes like C-type lectin domain members and perlucin-like protein. This continued up-regulation post-infection highlights their role in the immune response. Additionally, the leucine-rich repeat protein SHOC-2-like (SHOC-2) also showed sustained expression, with higher baseline levels in disease-resistant shrimp. SHOC-2 interacts with *Vibrio* flagellin A, activating Erk and STAT pathways, which induce ALF and C-type lectin expression, contributing to immunity^75^. Similar expression patterns of SHOC-2 and antimicrobial genes suggest comparable anti-*Vibrio* strategies in disease-resistant shrimp. However, few immune genes in susceptible shrimp matched those highly expressed in resistant ones, potentially explaining their vulnerability to *Vp*_HLVD_ infection.

## Conclusion

In our study, we discovered that disease-resistant shrimp exhibited effective combat against HLVD. Upon further investigation into the immune mechanism, we found that their disease resistance was attributed to their ability to counter *Vibrio*. Notably, even before infection, disease-resistant shrimp demonstrated a superior immune profile, characterized by an elevated baseline expression of immune-related genes. This inherent advantage significantly contributed to their capacity to effectively fight against *Vibrio* infections. Interestingly, upon *Vp*_HLVD_ infection, the immune response of disease-resistant shrimp exhibited a unique pattern. During the early stages of *Vp*_HLVD_ infection, their immune response was relatively slower compared to susceptible shrimp. However, as the infection progressed into the mid and late stages, their immune response intensified, showcasing a notable ‘lag’ phenomenon. Moreover, we identified several key genes crucial for *Vibrio* immunity, including serine protease inhibitor I/II-like, Kruppel homolog 1-like, ficolin-1-like, granulins-like, chitinase-3-like protein, anti-lipopolysaccharide factor-like, ctenidin-1-like, and perlucin-like protein. Additionally, we observed the involvement of the Toll/IMD signaling pathway, Apoptosis, and FoxO signaling pathway, as well as Lysosome activity, throughout the infection process in both shrimp populations. The identification of these potential genes and pathways is highly significant and will provide crucial guidance for future studies to explore shrimp defense mechanisms against *Vibrio*.

## Electronic supplementary material

Below is the link to the electronic supplementary material.

Supplementary Material 1

## Data Availability

The raw sequencing data have been submitted to the Short Read Archive (SRA) of NCBI under accession number SRR31440775-SRR31440786 with Bio project number PRJNA804687 (https://www.ncbi.nlm.nih.gov/bioproject/PRJNA1189178). All data generated or analyzed during this study are included in this published article (and its supplementary information files).
