# Supplementary material for: Transcriptomic insights into the resistance mechanism of Penaeus vannamei against highly lethal Vibrio parahaemolyticus
Source: Sci Rep. 2025 Apr 18;15:13490. doi: 10.1038/s41598-025-96168-3 (PMC12008197; doi:10.1038/s41598-025-96168-3)
Supplement: Supplementary file 1 — Supplementary Material 1 [file 41598_2025_96168_MOESM1_ESM.docx]

Table S1 Primers used for qRT-PCR analysis of DEGs and Tc toxin genes.

| Gene name | Primer name | Sequences (5′-3′) |
| --- | --- | --- |
| 16S rRNA | 16S-F | AGAGTTTGATCCTGGCTCAG |
|  | 16S-R | TGCTGCCTCCCGTAGGAGT |
| TcA | TcA-F | TCAGCCAAGCCTTAGGTC |
|  | TcA-R | TCAAACATTCACTGATCGCTTC |
| TcB | TcB-F | TGAGAGCTTTACGGTCCATG |
|  | TcB-R | ACGGATGACTCAATTAACCAAAC |
| Lv40S | Lv40S-F | TTGTGGAAGCGGCTGCTGTCAAG |
|  | Lv40S-R | AGAGGATCGTGGGAAGCGAGGTG |
| anti-lipopolysaccharide factor-like | LOC113810108-F | GCGAACAAACTCACTGGA |
|  | LOC113810108-R | ATGCGACCCCTGAAATAC |
| lysozyme-like | LOC113805933-F | CGACCGATTACTGGCTAC |
|  | LOC113805933-R | TTGCTGCGACCACATT |
| C-type lectin domain family 6 member A-like | LOC113812976-F | CTATTGTTGCTATGAGGATG |
|  | LOC113812976-R | GGTTGTATGTGGCGTTC |
| perlucin-like protein | LOC113824920-F | AACGGACACGAAGGAGACT |
|  | LOC113824920-R | CAAGAACCGAACACGAGAA |
| anti-lipopolysaccharide factor-like | LOC113820510-F | AGGCTTCCGAGCAACAC |
|  | LOC113820510-R | GAGCAATCAGGGCGAGT |
| leucine-rich repeat protein SHOC-2-like | LOC113812696-F | GAGAACGAACTCGCATTA |
|  | LOC113812696-R | GAACGACCTGGGACTGT |
| ctenidin-1-like | LOC113801835-F | AGGAAGAGGCGGAGTT |
|  | LOC113801835-R | AAGCCGTTGTTGAAGC |
| lysozyme-like | LOC113805418-F | TGGGACTGTAACGAAGC |
|  | LOC113805418-R | GTGTAGGAGCATGTATTTGA |
| C-type lectin domain family 7 member A-like | LOC113812977-F | TTACTCGTGCCCACTATCAG |
|  | LOC113812977-R | CCCCAAGAATCATTTCCTAT |
| peroxidase-like | LOC113807934-F | GCCGTCTTCTCATTCATTTG |
|  | LOC113807934-R | ACCCCACTTTACTCCACCCT |
| glutathione peroxidase-like | LOC113826359-F | CCGTTCTTGTCGATGAGGA |
|  | LOC113826359-R | AGCAGGAGCCAGGGAGTAA |

Table S2 Summary of sequencing data quality for transcriptome samples.

| Groups | Samples | Clean reads | Clean bases | Error rate | Q20(%) | Q30(%) | GC(%) |
| --- | --- | --- | --- | --- | --- | --- | --- |
| I 0 h | C3_0h_1 | 40790366 | 6.12 G | 0.03 | 97.12 | 92.60 | 49.99 |
|  | C3_0h_2 | 41586510 | 6.24 G | 0.03 | 97.15 | 92.66 | 48.81 |
|  | C3_0h_3 | 38794582 | 5.82 G | 0.03 | 97.06 | 92.66 | 51.14 |
|  | S_0h_1 | 40838512 | 6.13 G | 0.03 | 96.99 | 92.55 | 50.69 |
|  | S_0h_2 | 41356200 | 6.20 G | 0.03 | 97.34 | 93.10 | 48.95 |
|  | S_0h_3 | 41596952 | 6.24 G | 0.03 | 97.29 | 93.01 | 49.59 |
| I 6 h | C3_6h_1 | 40867336 | 6.13 G | 0.03 | 97.30 | 93.13 | 49.78 |
|  | C3_6h_2 | 40741978 | 6.11 G | 0.03 | 97.51 | 93.41 | 50.10 |
|  | C3_6h_3 | 40021010 | 6.00 G | 0.03 | 97.01 | 92.54 | 50.20 |
|  | S_6h_1 | 41193058 | 6.18 G | 0.03 | 97.17 | 92.86 | 50.59 |
|  | S_6h_2 | 40163516 | 6.02 G | 0.03 | 97.33 | 93.13 | 50.58 |
|  | S_6h_3 | 41063660 | 6.16 G | 0.03 | 97.42 | 93.32 | 50.06 |
| I 12 h | C3_12h_1 | 40148610 | 6.02 G | 0.03 | 97.41 | 93.24 | 50.11 |
|  | C3_12h_2 | 40460686 | 6.07 G | 0.03 | 97.40 | 93.20 | 50.27 |
|  | C3_12h_3 | 40769710 | 6.12 G | 0.03 | 97.36 | 93.10 | 49.11 |
|  | S_12h_1 | 40402330 | 6.06 G | 0.03 | 96.90 | 92.28 | 50.11 |
|  | S_12h_2 | 41236038 | 6.19 G | 0.03 | 97.36 | 93.15 | 50.06 |
|  | S_12h_3 | 40546052 | 6.08 G | 0.03 | 97.20 | 92.92 | 52.11 |
| A 0 h | B13_0h_1 | 41271632 | 6.19 G | 0.03 | 97.26 | 92.82 | 48.71 |
|  | B13_0h_2 | 39064740 | 5.86 G | 0.03 | 97.11 | 92.60 | 49.14 |
|  | B13_0h_3 | 40980286 | 6.15 G | 0.03 | 97.37 | 93.25 | 49.63 |
|  | B20_0h_1 | 39144858 | 5.87 G | 0.03 | 97.24 | 92.87 | 49.70 |
|  | B20_0h_2 | 41015578 | 6.15 G | 0.03 | 97.35 | 93.14 | 49.81 |
|  | B20_0h_3 | 42896544 | 6.43 G | 0.03 | 97.34 | 93.09 | 49.03 |
| A 6 h | B13_6h_1 | 41382840 | 6.21 G | 0.03 | 97.36 | 93.09 | 49.09 |
|  | B13_6h_2 | 42303092 | 6.35 G | 0.03 | 97.45 | 93.25 | 49.17 |
|  | B13_6h_3 | 41656270 | 6.25 G | 0.03 | 97.23 | 92.88 | 50.49 |
|  | B20_6h_1 | 38583570 | 5.79 G | 0.03 | 97.40 | 93.13 | 49.75 |
|  | B20_6h_2 | 42435596 | 6.37 G | 0.03 | 97.32 | 92.94 | 48.86 |
|  | B20_6h_3 | 40864808 | 6.13 G | 0.03 | 97.23 | 92.84 | 49.87 |
| A 12 h | B13_12h_1 | 40720848 | 6.11 G | 0.03 | 97.38 | 93.29 | 50.49 |
|  | B13_12h_2 | 40323822 | 6.05 G | 0.03 | 97.43 | 93.36 | 49.68 |
|  | B13_12h_3 | 41126602 | 6.17 G | 0.03 | 97.33 | 93.12 | 50.07 |
|  | B20_12h_1 | 52505320 | 7.88 G | 0.03 | 97.27 | 92.98 | 49.68 |
|  | B20_12h_2 | 42704228 | 6.41 G | 0.03 | 96.99 | 92.44 | 50.33 |
|  | B20_12h_3 | 40876922 | 6.13 G | 0.03 | 97.29 | 92.98 | 50.67 |

Note: I is the susceptible shrimp group; A is the disease-resistant shrimp group.
